# Effect of the in vivo application of granulocyte colony‐stimulating factor on NK cells in bone marrow and peripheral blood

**DOI:** 10.1111/jcmm.13539

**Published:** 2018-03-25

**Authors:** Xing‐Xing Yu, Ting‐Ting Han, Ling‐Ling Xu, Ying‐Jun Chang, Xiao‐Jun Huang, Xiang‐Yu Zhao

**Affiliations:** ^1^ Peking University People's Hospital Peking University Institute of Hematology Beijing Key Laboratory of Hematopoietic Stem Cell Transplantation Beijing China; ^2^ Peking‐Tsinghua Center for Life Sciences Beijing China; ^3^ Yantai YuHuangDing Hospital Yantai Shandong Province China; ^4^ Beijing Engineering Lab for Cell Therapy Beijing China

**Keywords:** GBM, G‐CSF, GPB, NGBM, NGPB, NK1, NK2, NK3, NKr

## Abstract

Granulocyte colony‐stimulating factor (G‐CSF) has been widely used in the field of allogeneic haematopoietic stem cell transplantation (allo‐HSCT) for priming donor stem cells from the bone marrow (BM) to peripheral blood (PB) to collect stem cells more conveniently. Donor‐derived natural killer (NK) cells have important antitumour functions and immune regulatory roles post‐allo‐HSCT. The aim of this study was to evaluate the effect of G‐CSF on donors' NK cells in BM and PB. The percentage of NK cells among nuclear cells and lymphocyte was significantly decreased and led to increased ratio of T and NK cells in BM and PB post‐G‐CSF in vivo application. Relative expansion of CD56^bri^ NK cells led to a decreased ratio of CD56^dim^ and CD56^bri^ NK subsets in BM and PB post‐G‐CSF in vivo application. The expression of CD62L, CD54, CD94, NKP30 and CXCR4 on NK cells was significantly increased in PB after G‐CSF treatment. G‐CSF treatment decreased the IFN‐γ‐secreting NK population (NK1) dramatically in BM and PB, but increased the IL‐13‐secreting NK (NK2), TGF‐β‐secreting NK (NK3) and IL‐10‐secreting NK (NKr) populations significantly in BM. Clinical data demonstrated that higher doses of NK1 infused into the allograft correlated with an increased incidence of chronic graft‐vs‐host disease post‐transplantation. Taken together, our results show that the in vivo application of G‐CSF can modulate NK subpopulations, leading to an increased ratio of T and NK cells and decreased ratio of CD56^dim^ and CD56^bri^ NK cells as well as decreased NK1 populations in both PB and BM.

## 1 INTRODUCTION

Allogeneic haematopoietic stem cell transplantation (allo‐HSCT) remains one of the most important curative methods for malignant haematologic diseases. However, its broad application is limited by the high incidence of graft‐vs‐host disease (GVHD). Current allo‐HSCT procedures consist mostly of bone marrow (BM) cells or granulocyte colony‐stimulating factor (G‐CSF)‐primed peripheral blood stem cells (GPB) or G‐CSF‐primed bone marrow (GBM). Natural killer (NK) cells mediate the early, non ‐adaptive responses against viruses and intracellular bacteria and modulate the activity of effector cells within the adaptive and innate immune systems.^1^ NK cells mediate these effects through the production of cytokines and the direct killing of transformed or infected cells.^2^, ^3^, ^4^ The function of NK cells is modulated by the balance of expression of inhibitory killer immunoglobulin‐like receptors (KIRs) and activating receptors on NK cells. Previous studies have shown that donor and recipient KIR ligand mismatch can initiate donor NK cell alloreactivity, leading to decreased leukaemia relapse and decreased GVHD incidence post‐haploidentical transplantation with T cell depletion in vitro.^5^ However, the predictive value of KIR ligand mismatch on clinical outcome has been inconsistent among different transplantation centres utilizing different protocols.^6^, ^7^ One of the most important reasons would be the in vivo application of G‐CSF for donor stem cell preparation that decreased the cytotoxicity of NK cells.^8^, ^9^ However, previous studies have considered only the cytotoxic roles of NK cells under haploidentical transplantation while neglecting the immune regulatory effect of NK cells.

Although both GBM and GPB contain large numbers of mature donor T cells that could cause GVHD,^10^ clinical data have demonstrated that the cumulative incidence of acute GVHD is acceptable with these cell sources.^11^ Based on the cytokine secretion model, CD4^+^ T cells can be classified as type 1 (Th1), type 2 (Th2) and type 17 (Th17) subpopulations of T cells.^12^, ^13^, ^14^, ^15^, ^16^, ^17^ Similar to Th1 and Th2 cells, human NK cells cultured in the presence of IL‐12 or IL‐4 can differentiate into populations with distinct patterns of cytokine secretion.^18^, ^19^, ^20^, ^21^, ^22^, ^23^ Specifically, IFN‐γ‐secreting NK cells (NK1) were shown to be important for infection control,^24^, ^25^ while IL13‐secreting NK cells (NK2), which contribute to IgE production by B cells, participate in the regulation of allergic airway responses.^21^, ^22^, ^23^ In addition, peripheral blood NK cells producing TGF‐β (NK3) and IL‐10 (NKr) have been shown to have a regulatory function in humans.^26^, ^27^, ^28^, ^29^ Our previous work demonstrated that the in vivo application of G‐CSF induced T cell hyporesponsiveness. In particular, the levels of Th1 and Th17 cells were decreased, while those of Th2 cells were increased in BM and PB grafts using G‐CSF.^13^, ^14^, ^15^, ^30^ However, the content and function of NK cells in BM before and after G‐CSF in vivo application have not been analysed.

The aim of this study was to explore the effect of G‐CSF on the NK1/NK2/NK3/NKr subpopulations, including CD56^bri^ and CD56^dim^ NK subsets, as well as the proliferation and cytotoxicity of NK cells in BM and PB. We also assessed the predictive roles of NK1, NK2, NK3 and NKr cells in allografts on clinical outcome post‐allo‐HSCT.

## 2 METHODS

### 2.1 G‐CSF treatment of healthy donors and sample collection

Steady‐state peripheral blood (NGPB) and bone marrow (NGBM) before G‐CSF in vivo treatment, as well as GBM and GPB, were obtained from 15 allogeneic donors. This group of donors, 8 men and 7 women, provided informed consent and had a median age of 29 years ranging from 18 to 54 years. Donors received recombinant G‐CSF (filgrastim; Kirin Co., Ltd., Tokyo, Japan) at a dosage of 5 μg/kg/d for 5 consecutive days. GBM was collected on the 4th day of treatment by aspiration, and GPB was obtained on the 5th day by leukapheresis using a continuous‐flow blood cell separator (Gambro BCT, Lakewood, CO, USA; or Baxter, Chicago, IL, USA). The reason for using this protocol was that patients in our institute receive transplants composed of GBM plus GPB, which are harvested on days 4 and 5, respectively.^31^, ^32^

Additional donors were collected GBM and GPB only. Therefore, totally 40 patients undergoing haploidentical (n = 27) and human leucocyte antigen (HLA)‐identical sibling (n = 13) allo‐HSCT between August 2009 and December 2009 were enrolled to explore the association of dose of NK1/NK2/NK3/NKr cells infused in allografts with clinical outcome post‐transplantation. The characteristics of the patients are shown in Table 1. All patients and donors provided written informed consent, and the Institutional Review Board of the Peking University Institute of Hematology approved the study.

**Table 1:** Patient and donor characteristics

Characteristics	Low NK1 group	High NK1 group	*P* value
No. of patient	18	22	
Median age (range), years	22 (11‐45)	32 (6‐46)	.286
Patient sex, male, No. (%)	11 (61.1%)	12 (54.5%)	.676
Donor‐recipient gender, No. (%)			.481
Male to male	4 (22.2%)	8 (36.4%)	
Male to female	5 (27.8%)	4 (18.2%)	
Female to male	6 (33.3%)	4 (18.2%)	
Female to female	3 (16.7%)	6 (27.3%)	
Diagnosis, No.			.28
AML	6 (33.3%)	12 (54.5%)	
CML	3 (16.7%)	5 (22.7%)	
ALL	8 (44.4%)	5 (22.7%)	
AA	1 (5.6%)	0	
Disease status, high risk, No. (%)	4 (23.5%)	4 (18.2%)	.682
Conditioning regimen, NO.			
Bu/Cy+ATG	18 (100%)	22 (100%)	
HLA‐A, HLA‐B, HLA‐DR mismatched grafts, No.			.761
0	7 (38.9%)	6 (27.3%)	
1	1 (5.6%)	3 (13.6%)	
2	4 (22.2%)	6 (27.3%)	
3	6 (33.3%)	7 (31.8%)	
ABO‐matched grafts, No. (%)			.54
Matched	9 (50%)	12 (54.5%)	
Major mismatch	5 (27.8%)	4 (18.2%)	
Minor mismatch	4 (22.2%)	4 (18.2%)	
Bidirectional mismatch	0 (0%)	2 (9.1%)	
Cell composition in allografts, median (range)			.527
Infused CD3+ cells, 10^8^/kg	1.7 (0.6‐3.3)	2.3 (0.4‐3.8)	
Infused NK1 cells,10^6^/kg	3.1 (1.0‐4.7)	8.0 (4.9‐36.2)	<.0001
Infused NK2 cells,10^5^/kg	2.7 (0.4‐2.0)	4.0 (0.2‐2.1)	.581
Infused NK3 cells,10^5^/kg	2.8 (0.52‐3.0)	4.9 (0.6‐2.3)	.112
Infused NKr cells,10^5^/kg	6.0 (0.11‐2.31)	1.6 (0.38‐7.2)	.101

### 2.2 Immunophenotyping, intracellular staining and multiparameter flow cytometric analysis

The monoclonal antibodies CD3‐PerCP, CD56‐allophycocyanin(APC), CD158a‐FITC, CD158b‐PE, CD158e‐FITC, CD94‐FITC, CD62L‐FITC, CD54‐PE, CD11a‐FITC, CX3CR1‐FITC, CXCR4‐PE, CCR7‐PE, NKP30‐FITC, NKP46‐PE, IL13‐PE, IL10‐PE, TGF‐β‐PE and IFN‐γ‐FITC (BD Bioscience, Mountain View, CA, USA), and NKG2A‐PE (BeckmanCoulter, USA) and appropriate isotypes were used in individual 4‐colour flow cytometry assays to analyse the immunophenotype as well as the cytokine secretion of NK cells. Intracellular staining was performed using the Pharmingen Intracellular Staining Kit (BD Pharmingen, San Diego, CA, USA). The cells were incubated for 5 hours with phorbol myristate acetate (PMA) (40 ng/mL) plus ionomycin (2.5 μg/mL, all reagents from Sigma Chemical) to stimulate maximal IFN‐γ, IL‐13, TGF‐β and IL‐10 production; GolgiStop (0.7 μL/mL) was added to the sample during the last 4 hours to trap the protein in the cytoplasm. NK1, NK2, NK3 and NKr cells were identified as CD3^−^CD56^+^IFN‐γ^+^, CD3^−^CD56^+^IL‐13^+^,CD3^−^CD56^+^ TGF‐β^+^ and CD3^−^CD56^+^IL‐10^+^, respectively. The dose of NK1, NK2, NK3 and NKr cells was classified as the absolute number of NK1, NK2, NK3 and NKr cells infused in GBM and GPB (cells/kg). Data were analysed using a FACSCaliber 4‐colour flow cytometer (BD Biosciences) and FlowJo 7.6.1 software (Tree Star Inc, CA, USA).

### 2.3 NK cell ex vivo proliferation assay and cytotoxicity assay

PBMCs collected from NGPB, GPB, NGBM or GBM were labelled with CellTrace CFSE Cell Proliferation Dye (Invitrogen) and placed in culture medium supplemented with IL‐15 (20 ng/mL; R&D Systems) for 7 days, followed by surface staining to test the proliferation of NK cells. Cytotoxicity of purified NK cells from NGPB, GPB, NGBM or GBM was tested by commercial cytotoxicity assays based on lactate dehydrogenase (LDH) detection according to the manufacturer's instructions (Cytotox 96; Promega, Madison, WI).^33^ Cytotoxicity of NK cells was examined using major histocompatibility complex (MHC) class I‐deficient human erythroleukaemia K562 cell line as targets, at effector‐target ratios ranging from 20:1 to 2.5:1. Meanwhile, PBMCs from NGPB, GPB, NGBM or GBM were also cultured in RPMI with 10% foetal calf serum and 1000 IU/mL interleukin 2 (IL‐2; Beijing Double‐Crane Pharmaceutical Co., Ltd) or IL15 (20 ng/mL; R&D Systems) for 10‐14 hours for both spontaneous, IL‐2‐stimulated or IL‐15‐stimulated NK cytotoxicity assays against K562 cell line at an effector‐to‐target ratio of 5:1 for 5 hours. GolgiStop (0.7 μL/mL) (BD Biosciences) were added after 1 hour. CD107a and IFN‐gamma production by NK cells was measured using the Pharmingen Intracellular Staining Kit (BD Pharmingen, San Diego, CA, USA).

### 2.4 Transplant procedure and definition

All patients received a myeloablative regimen, and conditioning was performed as previously described.^31^, ^32^, ^34^, ^35^ In HLA‐matched sibling transplants,^32^ patients received a regimen consisting of 80 mg/kg hydroxyurea orally on day 10, 2 g/m^2^/d cytarabine intravenously on day 9, 3.2 mg/kg/d busulfan intravenously on days 8 to 6 pre‐transplantation, 1.8 g/m^2^/d cyclophosphamide intravenously on days 5 to 4 pre‐transplantation and 250 mg/m^2^ of methyl‐N‐(2‐chloroethyl)‐N'‐cyclohexyl‐N‐nitrosourea orally on day 3. In HLA‐haploidentical donor transplants, patients received a regimen similar to HLA‐matched patients, except for the addition of 4 g/m^2^/d cytarabine on days 10 to 9 and 2.5 mg/kg/d antithymocyte globulin (SangStat) intravenously on days 5 to 2 pre‐transplantation. GVHD prophylaxis included cyclosporine A (CSA) and short‐term methotrexate (MTX) with mycophenolate mofetil (MMF).^31^, ^32^

### 2.5 Statistical analysis

To test differences in NK cell expression of receptors or cytokine secretion between NGPB and GPB; GPB and GBM; and NGBM and GBM, a Wilcoxon signed‐rank test or paired‐sample *t* test was used. Associations between the dose and percentage of NK1, NK2, NK3 and NKr cells infused in GBM or GPB and GVHD were calculated using cumulative incidence curves to accommodate competing risks. Gray's test was used in the cumulative incidence analyses.

## 3 RESULTS

### 3.1 Effect of G‐CSF on NK cell expansion

**Figure 1:**
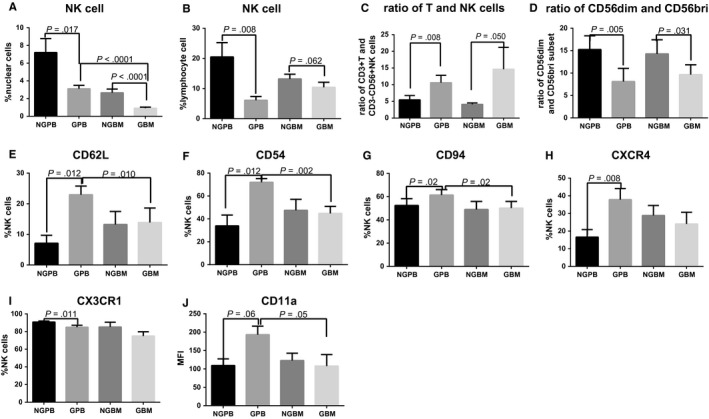
Comparison of NK cells between NGPB and GPB, NGBM and GBM, and GBM and GPB. A and B show comparisons of the percentage of NK cells among nuclear cells (A) and lymphoid cells (B) (n = 15); C and D show the comparison of the ratio of T and NK cells (C) as well as the ratio of CD56^dim^ and CD56^bright^ NK cells (D) (n = 15); E, F, G, H, I and J show comparisons of the expression of CD62L (E), CD54 (F), CD94 (G), CXCR4 (H), CX3CR1 (I) and CD11a (J) on NK cells (n = 9). The data are shown as the mean ± SEM of the indicated number of donors

The percentages of overall NK cells among nuclear cells and lymphoid cells were significantly decreased in BM and PB cells post‐G‐CSF in vivo application (*P* < .05, Figure 1A,B). The ratio of T cells and NK cells was significantly decreased in PB and BM after G‐CSF treatment (*P* < .05, Figure 1C). The relative expansion of the CD56^bri^ NK subsets led to a decreased ratio of CD56^dim^ and CD56^bri^ NK cells in GBM and GPB compared to that in NGBM and NGPB, respectively (*P* < .05, Figure 1D).

Considering that the expression levels of inhibitory receptors, activating receptors, adhesion molecules and chemokine receptors play important roles in regulating NK cell function, the expression levels of CD158a, CD158b, CD158e, CD94, NKG2A, CD62L, CD54, CD11a, CX3CR1, CXCR4, CCR7 and G‐CSFR on NK cells were evaluated before and after G‐CSF in vivo application. The expression levels of all tested molecules on NK cells in GBM were comparable to those in NGBM. The expression levels of CD158a, CD158b, CD158e, CCR7, NKP30, NKP46 and G‐CSFR on NK cells in NGPB were comparable to those in GPB. Meanwhile, these percentages of CD62L, CD54 and CD94 on NK cells in GPB were not only significantly increased compared with those on NK cells in NGPB but also increased compared to those on NK cells in GBM (Figure 1E‐G). The expression levels of CXCR4 on NK cells in GPB were only higher compared to those in NGPB (Figure 1H). In contrast,the expression of CX3CR1 on NK cells in GPB was significantly decreased compared to those in NGPB (Figure 1I). The MFI of CD62L, CD54, CD94 and CXCR4 on NK cells in GPB were also higher compared to those in NGPB (data not shown). The percentage of CD11a on NK cells was comparable among NGPB, GPB, NGBM and GBM, but the MFI of CD11a on NK cells in GPB had a trend to be higher compared to those in NGPB and GBM (Figure 1J).

The expression differences of CD158a, CD158b, CD158e, CD94, NKG2A, CD62L, CD54, CD11a, CX3CR1, CXCR4, CCR7 and G‐CSFR on CD56^bri^ or CD56^dim^ NK subpopulations among NGPB, GPB, NGBM and GBM were same to those on overall NK cells. The expression levels of CD158a, CD158b, CD158e, CD11a and CX3CR1 on CD56^bri^ subsets were lower than those on CD56^dim^ subset; however, the expression levels of CD94, CD62L, CD54, NKP30 and NKP46 on CD56^bri^ subset were higher than those on CD56^dim^ subsets (data not shown).

### 3.2 G‐CSF differentially affects NK cell subpopulations in BM compared to PB in vivo

**Figure 2:**
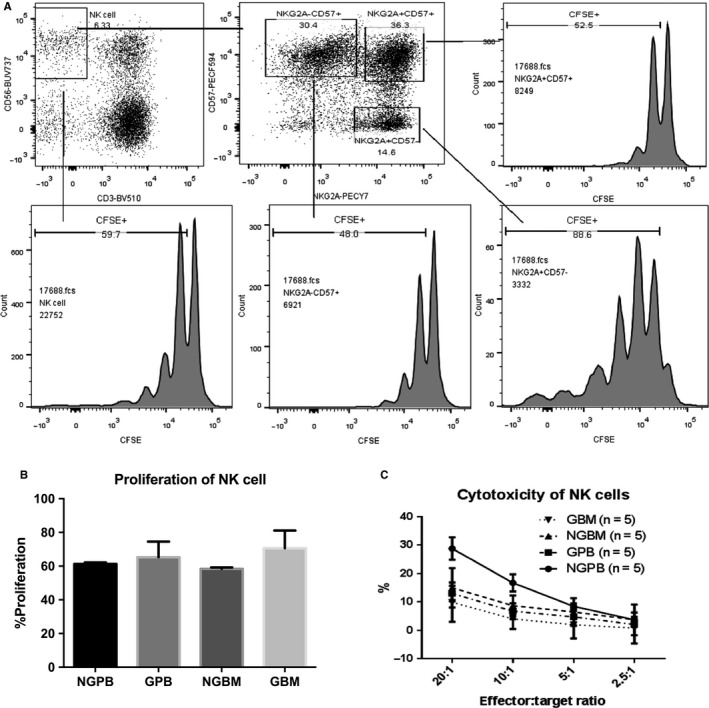
Comparison of proliferation of NK cells between NGPB and GPB, NGBM and GBM, and GBM and GPB. A shows representative flow cytometry plots of the proliferation of CFSE‐labelled NK cells, NKG2A^+^ CD57^−^ NK cells, NKG2A^−^ CD57^+^ NK cells and NKG2A^+^ CD57^+^ NK cells; B shows the comparison of the proliferation of NK cells among NGPB, GPB, NGBM and GBM (n = 3); C shows the comparison of the cytotoxicity of purified NK cells against K562 cells (n = 5). The data are shown as the mean ± SEM of the indicated number of donors

**Figure 3:**
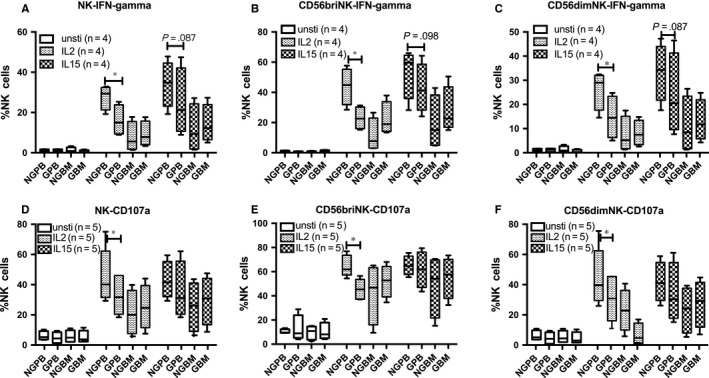
Comparison of IFN‐gamma and CD107a expression of NK cells against K562 cells among NGPB and GPB, NGBM and GBM. The expression of IFN‐gamma and CD107a of NK cells (A and D), CD56^bri^ NK cells (B and E), CD56^dim^ NK cells (C and F) against K562 cells without stimulation or with IL‐2 or IL‐15 stimulation among NGPB and GPB, NGBM and GBM

Cytotoxicity and proliferation capacity of NK cells were evaluated in BM and PB before and after G‐CSF in vivo application. No significant differences were found in the proliferation capacity of NK cells among NGPB, GPB, NGBM and GBM (Figure 2A‐B). Because NKG2A^+^CD57^−^, NKG2A^+^CD57^+^ as well as NKG2A^−^CD57^+^ NK subsets formed different development stages of CD56^dim^ NK cells,^36^ we further analysed the proliferation of NKG2A^+^CD57^−^, NKG2A^+^CD57^+^ as well as NKG2A^−^CD57^+^ NK cells to explore whether the different proliferation capacity of NK subsets could contribute to the decreased ratio of CD56^bri^ and CD56^dim^ in PB and BM after G‐CSF in vivo treatment. But no differences were found among the above‐mentioned NK subsets before and after G‐CSF in vivo application in PB and BM. Among the NKG2A‐ NK subpopulation, we further compared the proliferation capacity of licensed single KIR^+^ NK cells and unlicensed single KIR^+^ NK cells, and no significant differences were found (data not shown). As shown in Figure 1H, consistent with previous reports, the cytotoxicity of NK cells against K562 target cells was significantly decreased in GPB compared to that in NGPB; however, no significant difference was found in the cytotoxicity of NK cells between NGBM and GBM (Figure 2C). Meanwhile, the expression of IFN‐gamma and CD107a against K562 cells of overall NK cells, CD56^bri^ NK as well as CD56^dim^ NK cells had a trend to be decreased without cytokine stimulation or with IL15 stimulation in GPB compared to those in NGPB and was significantly decreased under the IL‐2 stimulation in GPB compared to those in NGPB (Figure 3A‐D). No differences were found in the expression of IFN‐gamma and CD107a against K562 cells of overall NK cells, CD56^bri^ NK as well as CD56^dim^ NK cells between NGBM and GBM (Figure 3A‐D).

**Figure 4:**
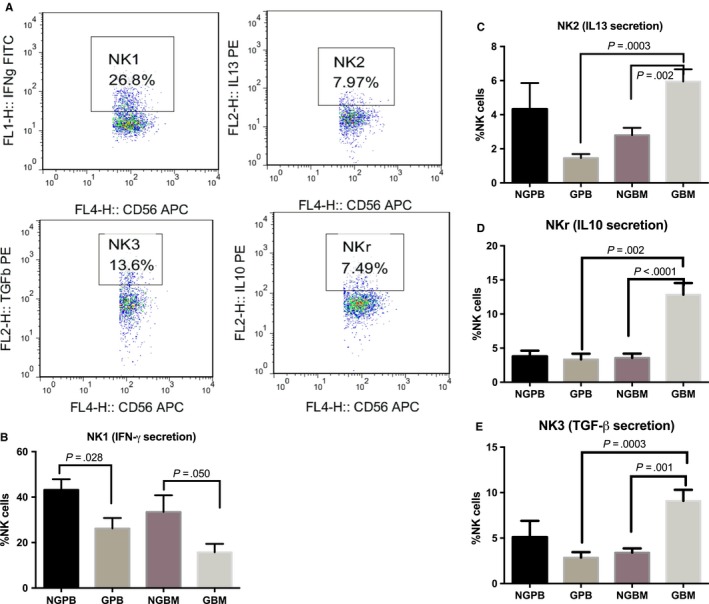
Comparison of NK subpopulations between NGPB and GPB, NGBM and GBM, and GBM and GPB. A shows the representative flow figures of NK1 cells, NK2 cells, NK3 cells and NKr cells among NK cells; B shows the comparison of the percentage of IFN‐γ‐secreting NK cells (NK1, n = 9); C shows the comparison of the percentage of IL13‐secreting NK cells (NK2, n = 9); D shows the comparison of the percentage of TGF‐β‐secreting NK cells (NK3, n = 9); E shows the comparison of the percentage of IL‐10‐secreting NK cells (NKr, n = 9). The data are shown as the mean ± SEM of the indicated number of donors

The percentage of the NK1 subset among NK cells was significantly decreased in either BM allografts or PB after the in vivo application of G‐CSF (Figure 4B). No significant differences were found in the percentages of the NK2, NK3 and NKr subsets between NGPB and GPB (Figure 3C‐E); however, the percentages of the NK2, NK3 and NKr subsets in GBM were significantly increased compared to those in NGBM or GPB, indicating the shift from NK1 to NK2/NK3/NKr subsets in BM after the in vivo application of G‐CSF(Figure 4).

We further analysed the secretion of IFN‐gamma, IL‐13, TGF‐beta and IL‐10 on CD56^bri^ and CD56^dim^ NK cells, respectively. Both of CD56^bri^ and CD56^dim^ NK cells could secrete IFN‐gamma, IL‐13, TGF‐beta and IL‐10. Secretion of IFN‐gamma was comparable between CD56^dim^ and CD56^bri^ NK subsets in NGPB,NGBM and GBM, but was higher in CD56^bri^ NK subsets compared to those in CD56^dim^ NK subsets among GPB (*P* = .017). Secretion of TGF‐beta was comparable between CD56^dim^ and CD56^bri^ NK subsets in NGBM and GBM, but was higher in CD56^dim^ NK subsets compared to those in CD56^bri^ NK subsets among NGPB and GPB (*P* = .05 and .01, respectively). Secretion of IL‐13 and IL‐10 was higher in CD56^dim^ NK subsets compared to those in CD56^bri^ NK subsets among NGPB and NGBM (*P* = .008 and .01 for IL‐13, *P* = .034 and .007 for IL‐10, respectively), but was comparable between CD56^dim^ and CD56^bri^ NK subsets in GPB and GBM.

### 3.3 Correlation between NK1\NK2\NK3\NKr cells in allografts with chronic GVHD occurrence post‐transplantation

**Figure 5:**
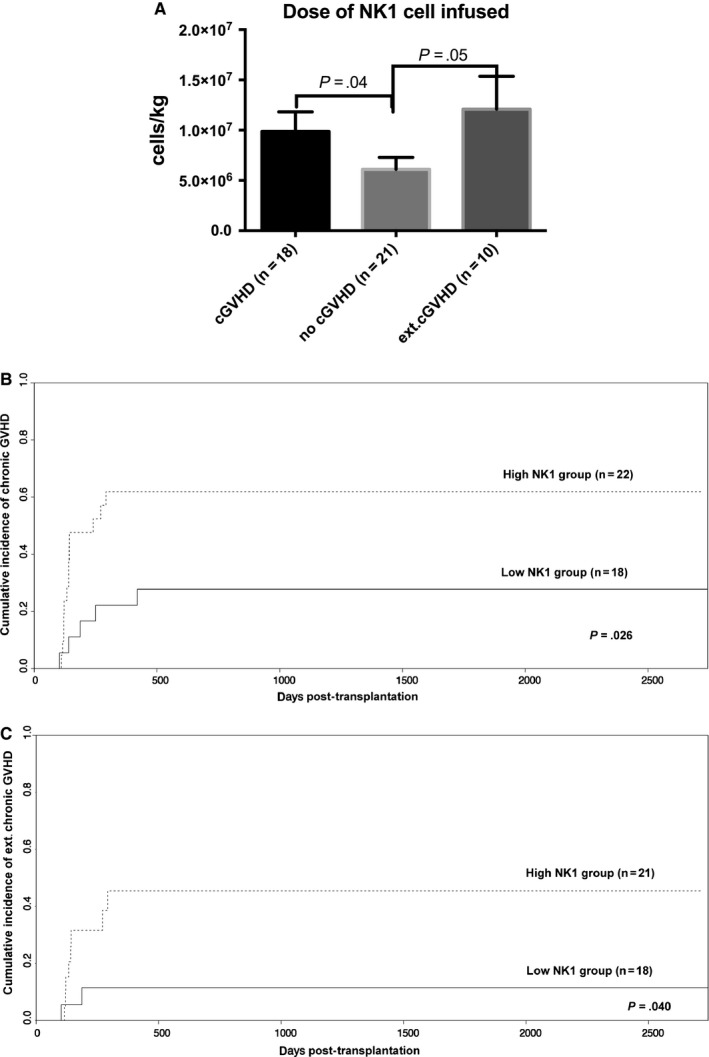
Correlation between the dose of NK1 cells infused and chronic GVHD. A shows the comparison of the dose of NK1 cells infused in allografts among patients without chronic GVHD (n = 21), with chronic GVHD (18) or with extensive chronic GVHD (n = 10); the estimated cumulative incidence rates of overall chronic GVHD (B) and extensive chronic GVHD (C) for patients in the low and high NK1 cell groups are separated according to the cut‐off number of NK1 cells in the allograft (4.8 × 10^6^/kg). The data shown are pooled from 41 experiments

Considering the decreased level of NK1 cells in GBM and GPB and the increased levels of NK2, NK3 and NKr cells in GBM, we further analysed the predictive value of NK subpopulations for clinical outcome. The dose of NK cells and the NK1/NK2/NK3/NKr subpopulations showed no association with the development of acute GVHD. However, patients enduring chronic GVHD (*P* = .040), especially extensive chronic GVHD (*P* = .050), post‐transplantation received a higher dose of NK1 cells in the allograft than those without chronic GVHD (Figure 5A). The dose of NK2/NK3/NKr cells, allograft T cells and IFN‐gamma secretion T cells infused in the GBM or GPB allograft was comparable in patients with and without chronic GVHD.

Based on the cut‐off value for the NK1 cell dose and chronic GVHD (4.8 × 10^6^/kg, sensitivity 72.2%, specificity 61.9%, *P* = .040), patients were subgrouped into either the high NK1 group (n = 21) or low NK1 group (n = 18). Patients in the high NK1 group demonstrated a higher cumulative incidence of overall cGVHD (61.91 ± 11.11% vs 27.78 ± 10.93%; *P* = .026; Figure 5B) and extensive cGVHD (45.41 ± 12.86% vs 11.46 ± 7.9%; *P* = .04; Figure 5C) than patients in the low NK1 group. The patient characteristics of the high and low NK1 groups (as shown in Table 1) were comparable, with the exception of the NK1 cell dose in the allografts. No associations were found between the NK subpopulation numbers in allografts and the relapse, treatment‐related mortality (TRM), overall survival (OS) and event‐free survival (EFS) rates (data not shown).

## 4 DISCUSSION

In this study, the following main findings were made: 1) in vivo application of G‐CSF not only decreased the percentage of NK cells but also modulated NK subpopulations, leading to an increased ratio of CD56^bri^ to CD56^dim^ subsets and low levels of NK1 cells, and 2) a high dose of NK1 cells infused into allografts correlated with the development of chronic GVHD after transplantation. The in vivo application of G‐CSF also led to expansion of NK2/NK3/NKr cells in GBM compared to those in NGBM and GPB; roles of NK2/NK3/NKr cells in allografts and clinical outcome post‐transplantation were still unclear.

A previous study demonstrated that myeloid progenitors were expanded, and lymphoid progenitors were decreased after the in vivo application of G‐CSF.^8^ Consistent with this previous report,^8^ the percentages of NK cells among GBM and GPB were significantly decreased in our study; therefore, the ratio of T and NK cells in GBM or GPB was significantly increased compared to that in NGBM and NGPB, respectively. However, we found that the ratio of CD56^bri^ to CD56^dim^ subsets was significantly increased in GBM and GPB compared to that in NGBM and NGPB, respectively, which differed from this previous report.^8^ Miller JS et al demonstrated that G‐CSF in vivo treatment had no effect on the proportion of CD56^bri^ NK cells.^8^ The reason for these disparate results could be because of the following two factors. First, different doses of G‐CSF were used for in vivo application; donors were treated with a daily dose of 5 μg/kg/d G‐CSF subcutaneously for 5 days in our centre, while a dose of 10 μg/kg/d G‐CSF subcutaneously for 5 days was used in the study by Miller JS et al. Second, the NGPB, GPB, NGBM and GBM were all collected from the same donor in this study, which increased the sensitivity to detect differences before and after G‐CSF treatment. However, Miller JS et al's report collected the GPB and NGPB samples from different donors. How G‐CSF could prompt the relative expansion of CD56^bri^ NK cells in vivo remains unknown. The G‐CSFR expression on NK cells was very low and was comparable before and after the in vivo application of G‐CSF in both PB and BM. Meanwhile, the proliferation capacities of NK cells induced by IL‐15 in vitro were also the same in NGPB and NGBM as in GPB or NGBM. CD56^bri^ NK cells have been shown to constitutively express the high‐ and intermediate‐affinity IL‐2 receptors and to expand in vitro and in vivo in response to low (picomolar) doses of IL‐2. These NK cells also express the c‐kit receptor tyrosine kinase, whose ligand enhances IL‐2‐induced proliferation.^37^ Meanwhile, most CD56^bri^ NK cells are present in lymph nodes. Therefore, G‐CSF in vivo treatment might induce CD56^bri^ NK cell expansion or redistribution through an indirect pathway. In addition, in accordance with the previous report, we confirmed that CD56^bri^ NK cells showed increased expression of CD94, NKP30, CD54 and CD62L, as well as lower expression of CX3CR1, KIRs, NKG2A and CD11a.^38^, ^39^ But, the expression differences of KIRs, CD94, NKG2A, CD62L, CD54, CD11a, CX3CR1, CXCR4, CCR7 and G‐CSFR on CD56^bri^ or CD56^dim^ NK subpopulations among NGPB, GPB, NGBM and GBM were same to those on overall NK cells. Therefore, the increased expression of CD62L, CD54, CD94, NKP30, CXCR4 and CD11a and decreased expression of CX3CR1 on NK cells in GPB could not solely be the expansion of CD56^bri^ NK cells. The underline mechanism was still unknown, but the increased levels of CD62L, CD54 and CXCR4 would be helpful for NK cells homing.

In addition, we found that G‐CSF in vivo application significantly decreased the levels of NK1 cells in GBM and GPB, but increased the levels of NK2, NK3 and NKr cells in GBM. The percentage of NK1/NK2/NK3/NKr on CD56^bri^ or CD56^dim^ NK subpopulations among NGPB, GPB, NGBM and GBM was same to those on overall NK cells. Both of CD56^bri^ and CD56^dim^ NK subpopulations had NK1/NK2/NK3/NKr subpopulations under the stimulation of PMA and ionomycin. The reason for this shift from NK1 to NK2/NK3/NKr cells remains unknown. Our previous study demonstrated that G‐CSF treatment dramatically decreased the quantities of Th1 cells.^14^, ^15^ The reason for the shift from Th1 to Th2 cells could be caused by the expansion of DC2 cells in vivo after G‐CSF application. Future work should investigate the effect of the DC2/DC1 balance on the cytokine secretion of NK cells. Meanwhile, consistent with the fact that Th1 cells can contribute to chronic GVHD progression,^40^ we found that a high dose of NK1 cells infused in the allografts correlated with an increased incidence of chronic GVHD post‐transplantation. NK cells have been traditionally recognized as possessing antitumour functions without causing GVHD.^41^ However, Shah NN et al reported that adoptive IL‐15/4‐1BBL‐activated NK cell infusion post‐HLA‐matched, T cell‐depleted transplantation contributed to acute GVHD.^42^ Therefore, the contribution of NK1 cells to the prognosis of chronic GVHD warrants further study.

Meanwhile, in accordance with a previous report,^8^, ^9^ we found that the cytotoxicity of overall NK cells, CD56^bri^ NK subsets as well as CD56^dim^ NK subsets in GPB was significantly decreased compared to that in NGPB, which could not be rescued by IL‐2 stimulation. Based on the data in this study, we propose the following hypotheses. Increased expression of CD94 on NK cells, but no significant change in the expression of KIRs and NKG2A, might contribute to the decreased cytotoxicity of NK cells in GPB compared to NGPB. Borrego et al^43^ demonstrated that the CD94 receptor can block CD69‐initiated cytotoxic effects of NK cells.

In summary, we demonstrate that the in vivo application of G‐CSF can modulate NK subpopulations, leading to a high ratio of CD56^bri^ to CD56^dim^ subsets and low levels of the NK1 population. A higher dose of NK1 cells infused in allografts correlated with an increased incidence of chronic GVHD after transplantation. Future work is aimed at investigating the molecular pathway underlying the effect of G‐CSF on NK cells, which would be helpful for decreasing GVHD but maintaining the potential antitumour benefits of NK cells in the allograft setting.

## CONFLICT OF INTERESTS

The authors declare that they have no Conflict of interests.

## AUTHORS' CONTRIBUTIONS

XY Zhao conducted the flow cytometry assays, performed the statistical analyses and drafted the manuscript. TT Han, Yu XX and Xu LL also conducted the flow cytometry assays and participated in data analyses. XJ Huang participated in the design of the study and critically revised the study for important intellectual content. All other authors were involved in the study design, the discussions and the treatment of patients at Peking University Institute of Hematology. All authors read and approved the final manuscript.
